# Breastfeeding practices and perspectives in the setting of maternal HIV in high-income countries since the shift in the United States national guidelines: a scoping review

**DOI:** 10.3389/frph.2026.1864784

**Published:** 2026-06-26

**Authors:** Naquia Unwala, Presley Simmons, C. Scott Dorris, Rachel K. Scott

**Affiliations:** 1 Georgetown University School of Medicine, Washington, DC, United States; 2 The Women's and Children's Research Network, MedStar Health Research Institute, Washington, DC, United States

**Keywords:** breast feeding, HIV, infant nutrition physiological phenomena, infectious disease transmission, vertical, postpartum period

## Abstract

**Introduction:**

United States national guidelines around infant feeding in the setting of maternal HIV were updated significantly in 2023. The updated guidelines encourage evidence-based counseling and emphasize shared decision-making around infant feeding, supplanting a blanket recommendation for replacement feeding. This scoping review seeks to capture the shift in clinical practices and perspectives of people living with HIV and their healthcare providers since 2023.

**Methods:**

Our review followed the Preferred Reporting Items for Systematic reviews and Meta-Analyses (PRISMA) extension for scoping reviews. We conducted a search of articles published in or after 2023 and focused on infant feeding guidelines in the setting of HIV or the infant feeding experiences of people living with HIV and providers that care for individuals with HIV in the peripartum and postpartum periods. We excluded studies that were not published in English. We searched MEDLINE, Embase, Cochrane's CENTRAL, CINAHL Ultimate, and Web of Science Core Collection and included all years from 2023 to the final date of search on January 6, 2026.

**Results:**

Our search yielded qualitative studies on both provider and patient experiences, protocols for implementation of the new guidelines, quantitative studies on HIV transmission in breastfeeding people living with HIV, and opinion pieces rooted in evidence. Our review found emergent safety data on breastfeeding among people living with HIV. Studies on provider experiences demonstrated variability in experience and comfort around breastfeeding, exacerbated by a lack of institutional guidelines on breastfeeding. Our review both highlighted evidence and expert opinion that counselling on infant feeding should be conducted by a multidisciplinary care team and tailored to the individual patient. Patient experiences highlighted that infant feeding decisions among people living with HIV are shaped by sociocultural contexts, personal motivations to breastfeed, patient knowledge on breastfeeding safety, and communication with their healthcare provider.

**Conclusions:**

Recommendations to optimize counseling and care around infant feeding include an approach that is evidence-based, culturally sensitive, and free from bias. Areas for continued work include research on the optimal frequency of maternal and infant testing in the postpartum period and efforts to implement infant feeding guidelines in a clear and consistent manner across institutions and healthcare practices.

## 1 Introduction

HIV viral particles (i.e., free virus and infected cells) can be transmitted through chest or breastmilk (subsequently referred to as breastmilk), particularly in the setting of elevated maternal viral load (VL) and in the absence of maternal antiretroviral therapy (ART). The risk of perinatal transmission of HIV through breastmilk is low (i.e., estimated to be less than 1%) in the context of consistent maternal ART use throughout pregnancy, sustained maternal viral suppression, and appropriate neonatal antiretroviral post exposure prophylaxis (1). Given the emphasis on shared decision-making around infant feeding in the United States (U.S.) Department of Health and Human Services (DHHS) Perinatal HIV Guidelines since 2023, providers in the U.S. have begun to see more open interest in breastfeeding among mothers or birthing parents (subsequently referred to as “mothers”) with HIV. There are limited data, however, on provider clinical practices around infant feeding in the setting of maternal HIV and on the infant feeding experiences of people living with HIV (PLWH) in the U.S. and other resource-rich, high-income countries.

Breastfeeding in the setting of maternal HIV has been an ongoing public health concern and topic debate for the duration of the HIV epidemic. In low-resource settings, the World Health Organization (WHO) and others public health guidelines have supported exclusive breastfeeding, even before the widespread availability of ART, due to evidence suggesting infant mortality risk from acquiring HIV via breastfeeding was outweighed by infant mortality risk from replacement feeding secondary to water-borne illnesses and malnutrition (2). In resource-rich settings, however, the risk of mother-to-child transmission (MTCT) of HIV through breastfeeding was believed to outweigh the benefits. Replacement feeding with donor milk or formula was therefore universally recommended by the CDC, the DHHS Perinatal Guidelines, and professional organizations such as the American Academy of Pediatrics (AAP) and the American College of Obstetricians and Gynecologists (3), creating a double standard for the global North and South. This push towards the zero-risk option, however, did not consider the overall wellbeing of the mother, as breastfeeding carries significant cultural, social, and psychological weight (4), nor of the neonate, given the numerous nutritional, developmental, and immunological benefits of breastfeeding (5–8).

The scientific impetus for the shift in guidelines and practices in the U.S. was the advent of data supporting the safety of breastfeeding in the setting of maternal ART and viral suppression. The PROMISE trial in 2014 (9) and the DolPHIN trial in 2022 (10) demonstrated that maternal ART and infant prophylaxis are safe, well tolerated, and significantly reduce MTCT through breastfeeding to three to six per thousand by 12 months of age. Additionally, a growing body of literature as captured in a review by Espinal et al. added additional support to the safety of breastfeeding in the setting of virally suppressed HIV (11).

In parallel to mounting data supporting the safety of breastfeeding in the setting of HIV, there were increasing calls from advocates and from the community (1) to shift the paradigm of paternalism and protectionism around infant feeding to patient-centered counseling and shared decision-making, and (2) to recognize that sustained maternal viral suppression substantially reduces the risk of transmission during breastfeeding. The updated DHHS perinatal guidelines for infant feeding recommendations for PLWH were published in early 2023, with an emphasis on evidence-based, patient-centered counseling about infant feeding options to enable shared decision-making between PLWH and their providers. The guidelines emphasize the overall safety of breastfeeding, specifically the results of the PROMISE trial demonstrating that breastfeeding in the setting of maternal viral suppression carries a risk of MTCT less than 1% (9).

The guidelines recommend coordination of care between the maternal care provider, infectious disease, pediatrics, social work, a breastfeeding specialist, and lactation support (1), and that lactation support specialists should be aware of the challenges faced by PLWH and the circumstances under which breastfeeding should be paused or discontinued. Specifically, the guidelines stipulate that if individuals are not virally suppressed or if VL becomes temporarily detectable, replacement feeding with formula milk or pasteurized human donor milk is recommended to eliminate the risk of MTCT. Continuing to ensure access to ART and screening/providing support for postpartum depression will maximize adherence and safety of breastfeeding. If individuals elect to replacement feed, the guidelines recommend exploring and addressing potential barriers to feeding with formula and/or pasteurized human donor milk (1).

This scoping review captures the published literature in the first three years since the U.S. guidelines update, specifically focusing on shifts in practice and perspectives in high-income, high-resource settings.

## 2 Methods

### 2.1 Design

This scoping review followed the Preferred Reporting Items for Systematic Reviews and Meta-Analyses (PRISMA) extension for scoping reviews (12).

### 2.2 Eligibility criteria

Eligible studies were published in or after 2023 and either focused on breastfeeding guidelines for PLWH or the experiences of PLWH/providers that care for PLWH. We excluded studies that were not published in English. Additionally, we excluded studies that were focused on MTCT and/or ART adherence *outside* of the context of breastfeeding.

### 2.3 Information sources

We searched MEDLINE, Embase, Cochrane's CENTRAL (all through Ovid), CINAHL Ultimate (EBSCOhost), and Web of Science Core Collection and included all years from 2023 to the final date of search on January 6, 2026.

### 2.4 Search

The systematic search was developed by an experienced health sciences librarian in collaboration with the rest of the team. We first tested the draft search in Ovid MEDLINE to ensure an optimal balance sensitivity vs. specificity. The search strategy consisted of a combination of a database-specific subject headings and keywords related to the concepts of HIV infections, breastfeeding or bottle feeding, and provider practice or patient preferences. Inclusive language (e.g., “birthing people”) was used in addition to “women” with HIV to fully capture the range of experiences that have been documented. To supplement the systematic searches, we conducted targeted hand searches to identify potentially relevant studies. We utilized those studies as seeds to conduct a supplementary search within Ovid MEDLINE to ensure that we were not missing any possible studies. Please see Supplementary Table 1 for full reproducible search strategies.

### 2.5 Selection of sources of evidence

Articles were uploaded to the Covidence platform and prior to screening, the system automatically identified and removed duplicate citations. Two independent reviewers conducted Screening of the Titles/Abstracts and Full Text, and any conflicts were resolved as a pair. Studies that were conducted in a low-income/low-resource area were excluded. This distinction was made based on the level of income of the country according to the World Bank Classification System for fiscal year 2025–2026. Two independent reviewers created a data extraction template within Covidence. They used this template to extract data and differences were reconciled as a pair.

### 2.6 Framework

The shared decision-making model of healthcare is a process in which healthcare providers and patients work together to bridge gaps between clinical evidence and the patient's personal and cultural values to make collaborative healthcare decisions (13). Inherent to the shared decision-making model is provider conference of agency to the patient via provision of education/information (e.g., reviewing the updated current infant feeding guidelines and safety data) and provider support of the decision-making process (e.g., eliciting and answering questions, providing counseling, respecting/supporting the infant feeding plan). We selected this framework as the 2023 DHHS shift in guidelines was both informed by and embodies this model. Across the studies examined in this review, the shift in the recommendations around infant feeding counseling for this population reflect broader themes related not only to shared decision-making, but also patient autonomy, reproductive justice, and the structural factors that influence infant feeding decision-making.

## 3 Results

**Figure 1:**
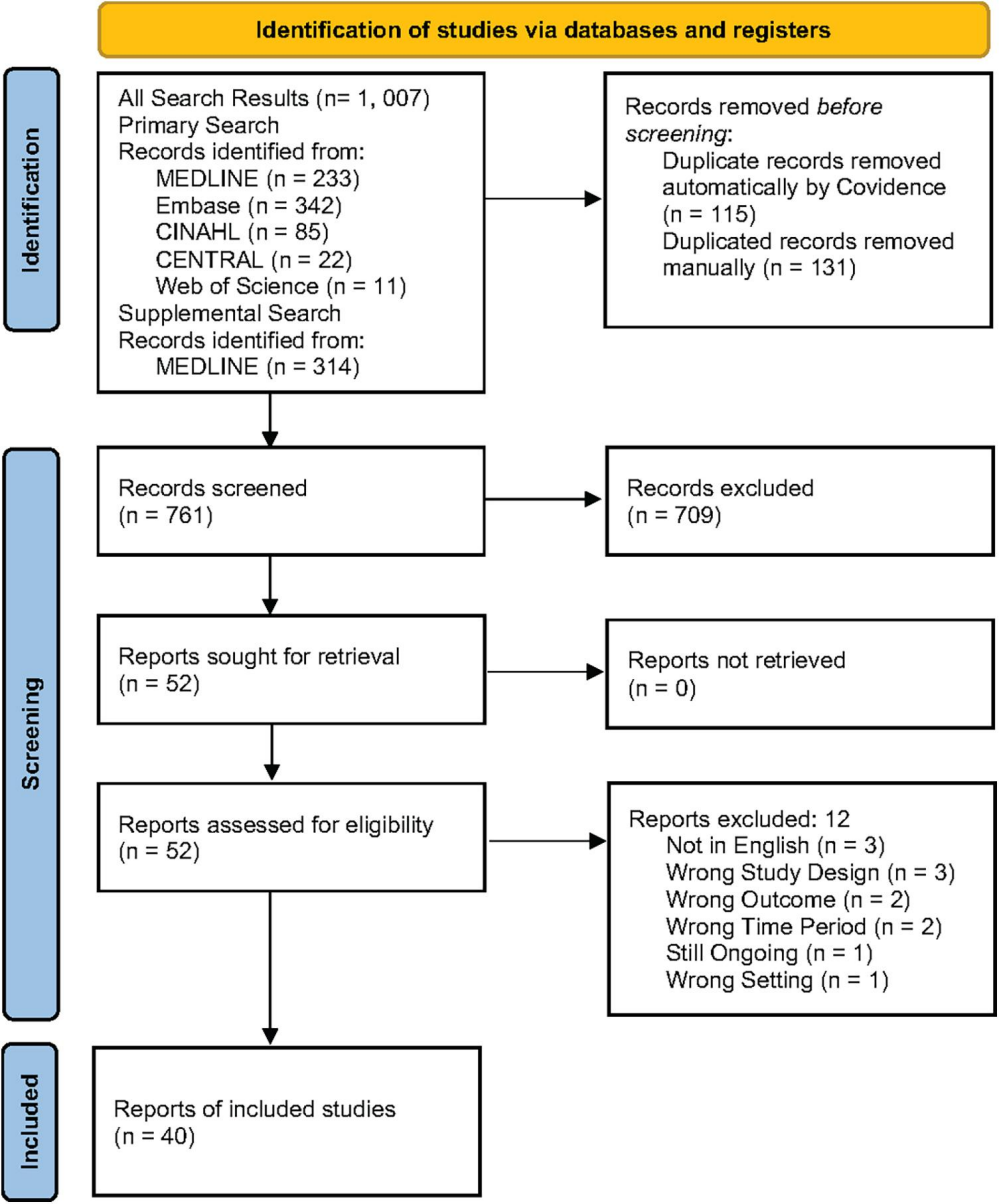
PRISMA flow diagram of study selection process.

Our literature search yielded 1,007 potential publications; Figure 1 depicts the PRISMA flow diagram. Of these, 246 were duplicates (115 automatically removed by Covidence, 131 removed by reviewers), which left us with 761 potential publications. After title and abstract review, 709 records were excluded after additional filters for articles that are not full text, based on non-human studies, and not identified as English articles. After title/abstract review, an additional 12 articles were excluded for reasons including exclusionary study design, exclusionary outcome, exclusionary time period, exclusionary setting, still on going, and not in English. After full-text review, we included 40 full-text articles primarily categorized as qualitative studies on both provider and patient experiences (*n* = 14), protocols for implementation of the new guidelines and surveys of current practices (*n* = 16), quantitative studies on HIV transmission in breastfeeding PLWH (*n* = 5), and opinion pieces rooted in evidence (*n* = 5). Supplementary Table 2 describes the included studies.

### 3.1 Safety of breastfeeding in virally suppressed PLWH in resource-rich countries

Since the 2023 shift in U.S. guidelines, data from multiple additional studies in resource-rich settings have further supported the safety of breastfeeding (Table 1).

**Table 1:** Safety data on breastfeeding in the setting of maternal HIV published since 2023 (14–17).

Location & Time period	Mothers with HIV (n)	Breastfed infants (n)	Neonates with conclusive negative testing (n)	Neonates awaiting confirmatory testing (n)	Neonates lost to follow-up (LTFU) (n)	Additional findings
Berlin, Germany 2017–2023	77	77	75	2	0	Region of origin: Sub-Saharan Africa-63% Western Europe-29% Eastern Europe/Russia-6% Initiation of ART prior to pregnancy Yes (*n* = 69) No (*n* = 8) Median duration of breastfeeding: 7.3 months (IQR=7.6, range=0.1 to 33.9) Duration of breastfeeding and mode of delivery were positively correlated (*p* = 0.005): SVD (52.6%); 10 months C-section (40.8%); 5 months Vacuum extraction (6.6%); 3–4 months
United Kingdom (UK) 2012–2021	201	203	150	34	5	170/201 mothers were from outside of the UK; 154 from Sub-Saharan Africa. Motivation for breastfeeding: Bonding (69%) Health benefits (60%) Disclosure concerns (26%) Previous BF since dx (26%) Family or friends’ expectations or pressure (22%)
Multi-site (U.S. and Canada) 2014–2022	72	72	68	0	4	Challenges to breastfeeding: Low milk supply (*n* = 15) Pain (*n* = 4) Mastitis (*n* = 3) Difficulty latching (*n* = 4) Cracked nipples (*n* = 3)
Wisconsin, U.S. 2016–2023	5	7	5	1	1 LTFU; subsequently tested positive	

These studies in resource-rich settings reinforce not only the safety of breastfeeding in mothers with virally suppressed HIV, but also the growing proportions of PLWH electing to breastfeeding in the context of growing safety data and patient-centered practice guidelines emphasizing shared decision-making around infant feeding. In Berlin, Feiterna-Sperling and colleagues described an increasing proportion of mothers electing to breastfeed each year between 2017 and 2023, attributed to “evolving counseling practices, greater patient awareness, and updated guidelines that support breastfeeding for [PLWH] under optimized conditions” (14). Francis and colleagues in the United Kingdom (UK) similarly noted a fourfold increase in the proportion of PLWH opting to breastfeed between 2012 and 2021 (15).

### 3.2 Domestic responses in practice guidelines and institutional practices following the DHHS guidelines shift

In the U.S., following the 2023 DHHS guideline shift several professional societies have released updated aligning guidelines. The AAP released an updated guideline emphasizing similar concepts of shared decision-making and patient autonomy (18). The AAP acknowledged potential exacerbation of existing health disparities for Black and other people of color, specifically that refraining from breastfeeding may compound an already higher risk of adverse health outcomes. While disproportionately impacted by HIV, Black non-Hispanic women also have the lowest breastfeeding rate of any racial or ethnic group in the U.S. (19). Systemic racism has led to both the inaccurate belief that Black women prefer to bottle feed rather than breastfeed, and to structural barriers which limit or prevent breastfeeding (e.g., inflexible, low-wage jobs, short maternity leaves, financial pressure, lack of cultural normalization, lack of support) (19). Similarly, the Association of Nurses in AIDS Care (ANAC) released a position statement in 2025 in support of person-centered counseling with an emphasis on relational decision-making, continuity of care, and trauma-informed practices (20).

In the U.S., several centers have published their institutional experiences prior to guidelines change (21) and their centers' approaches since the updated guidelines for infant feeding (17, 22, 23). Abuogi et al. describe how their experience at Children's Hospital Colorado Immunodeficiency Program prior to the 2023 guidelines change informed their center's protocols for breastfeeding among PLWH and shared decision model (21). Their program observed earlier cessation of breastfeeding among PLWH than in the general population and postulated that earlier support from the care team may have prolonged their patients' ability to continue breastfeeding. They additionally noted challenges including mastitis, low milk supply, spikes in plasma VL, and difficulty weaning. The authors describe learning from these complex clinical issues and how they shaped internal clinical protocols for management of common clinical scenarios, e.g., management of infant feeding in the setting of maternal VL >20 copies/mL, and development of educational materials for breastfeeding mothers to prepare them for potential breastfeeding challenges. Although the institutional approaches are unique, there are many commonalities in recommended approaches reviewed in Table 2.

**Table 2:** Commonalities noted in institutional guidelines/protocols (17, 21–23).

Theme	Key findings
Initial consultation re. infant feeding	Initiate discussion regarding infant feeding desires/intentions, rationales and knowledge using open-ended and non-judgmental questions Conduct an infant feeding needs assessment and exploration of potential barriers to remaining in HIV care/ART adherence Prior breastfeeding experience Risk factors for poor breastfeeding outcomes Duration of HIV diagnosis Social support system Goal is not to reach a decision on infant feeding, but to introduce topics that will be discussed in further detail later
Patient-centered counseling	Review evidence-based guidelines, including what is known and unknown about infant feeding Emphasize importance of maintaining viral suppression for wellbeing of mother and infant
Multidisciplinary team involvement	Engage and involve providers from pediatrics/neonatology, infectious disease, lactation, nursing, social work, legal, ethics, peer navigation for a multidisciplinary approach
Finalization of plan	Ensure entire multidisciplinary care team is aligned with the infant feeding plan Communication between the prenatal team and the planned pediatric team to ensure continuity of care
Postnatal care	Warm handoff from maternal care to infectious disease Close follow-up with infant’s pediatrician Continue to monitor maternal ART adherence and VL testing Monitoring of neonatal antiretroviral prophylaxis administration and nucleic acid testing

There is consensus that infant feeding counseling should be ongoing, evidence-based, and patient-centered (17, 21–23), with the goal of having an infant feeding plan in place by the third trimester (including postnatal follow up with the infant's pediatrician for HIV testing and the mother's obstetrician and/or infectious disease physician for continued VL monitoring. Notably, one site employs the use of a patient-physician breastfeeding agreement in which the patient initials next to statements such as “I understand that if I breastfeed, there is a small risk of transmitting HIV to my baby through breast milk” and “I will have a viral load checked as recommended at least every 2 months” (23). The agreement is not a legally binding contract, but rather a demonstration of support for the patient's infant feeding decision by the healthcare team (23). At other institutions, nurse navigators facilitate the postpartum continuity of care around infant feeding, including arranging consultation with a lactation specialist prior to hospital discharge and confirming that the infant is receiving postnatal antiretroviral prophylaxis (17). After discharge, the nurse navigator assists with both the maternal and neonatal medication adherence and retention in care, including appropriately-timed VL testing (17).

Several publications also describe their approaches to gaps in both the scientific literature and accordingly in the DHHS guideline recommendations. Notably, the DHHS guidelines acknowledge that there are no data to inform the frequency of maternal VL monitoring (1, 24), and recommend testing every one to two months, based upon observational data and expert opinion (16, 17, 25). In mothers who are breastfeeding, ART adherence support and monitoring of maternal VL remains paramount as ART adherence (and by extension maternal VL) are not infrequently impacted by postpartum depression (PPD), in addition to the responsibilities of childcare and an altered sleep schedule (26). In clinical practice, Powell et al. describe testing monthly; McKinney et al. describe initially testing at two and six weeks postpartum followed by testing every two months (22, 23) Although there is consensus that postpartum testing should take place more frequently than the 3-month intervals at which VL is typically tested, an evidence-based time interval for VL monitoring has yet to be determined (27). Of note, some experts caution of the risk of over-medicalizing the postpartum period and associated risk of increased maternal anxiety (28).

Taken together, the experiences and care models put forth by these centers and topical experts highlight the importance of early assessment of infant feeding desires and implementation of multidisciplinary support, closer regular follow-up while breastfeeding, and active management of breastfeeding challenges, such as mastitis and elevated maternal VL. It is also important to remain cognizant of additional challenges affecting PLWH that may impact both maternal ART adherence and viral suppression and breastfeeding, such as elevated risk of PPD, systemic racism, and historic disenfranchisement.

### 3.3 Infant feeding guidelines and practices in other high-income countries

**Figure 2:**
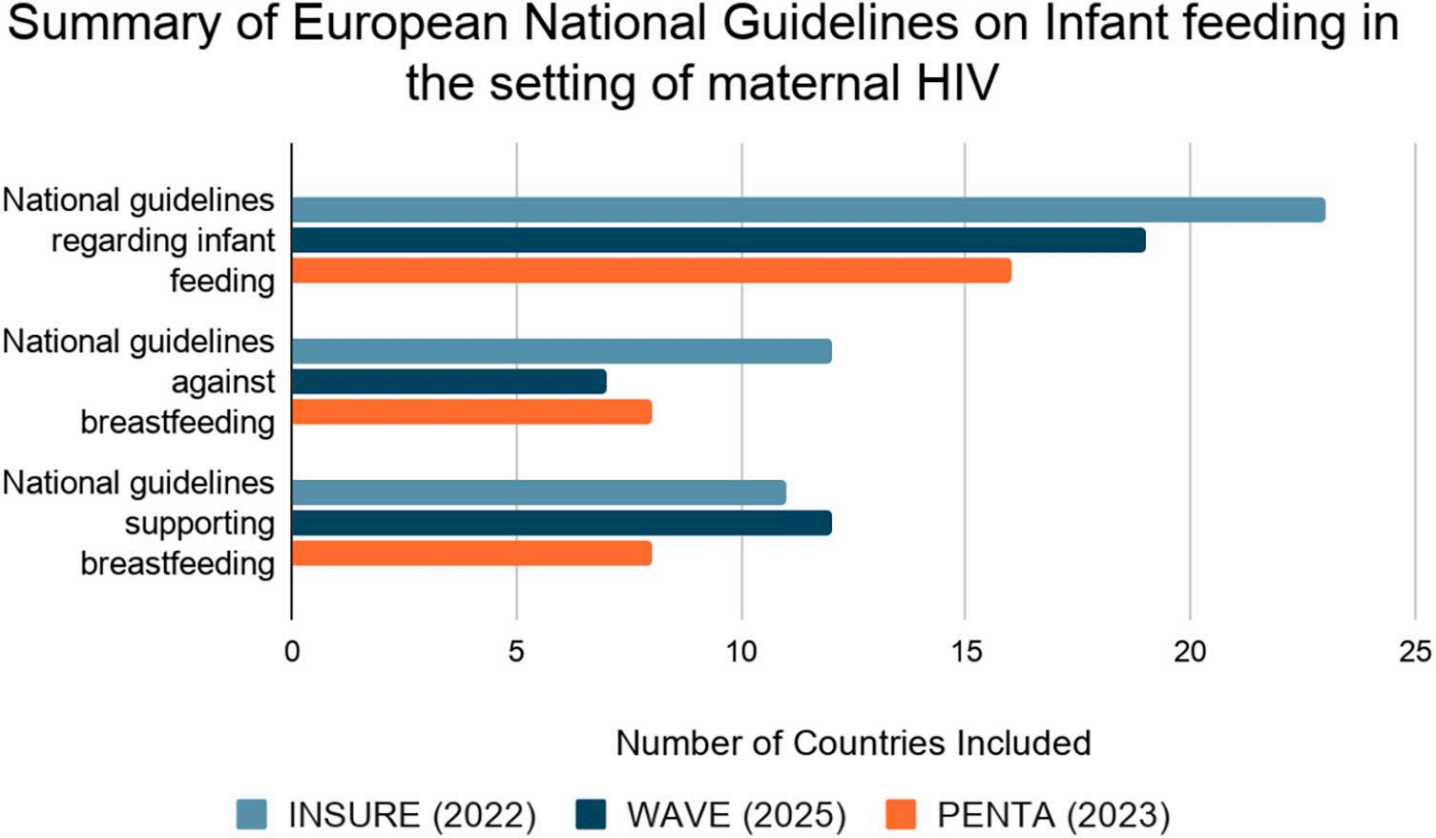
Summary of European national guidelines on infant feeding in the setting of maternal HIV.

Several publications describe infant feeding guidelines across Europe (Figure 2).

In association with the Women Against Viruses in Europe (WAVE), Keane et al. conducted the HIV aNd BreaStfeeding in EUropE (INSURE) survey in 2022 to collate breastfeeding trends, practice, and guideline recommendations for PLWH across Europe (29). They subsequently published a 2025 review (the WAVE survey), which examined national European guidelines of infant feeding in the setting of maternal HIV with the goal of consolidation in order to better inform the decision-making process (30).The WAVE survey found that a subset of guidelines explicitly address the challenges and benefits of breastfeeding including “the emotional, financial, and social costs of not breastfeeding” (UK), issues with more intense monitoring in the postpartum period (Switzerland), discrimination against PLWH who are not breastfeeding (Germany, Austria), and the well-known benefits of breastfeeding for both the lactating mother and the infant (Switzerland, Germany, Austria) (30) The majority of guidelines recommended monthly VL testing postpartum for breastfeeding mothers, with minor deviations (i.e., Belgium recommends testing every 6 weeks, Turkey recommends testing every 1–2 months). Most recommend against mixed feeding as it potentially increases risk of HIV transmission, but the UK guidelines specify conditions under which mixed feeding/formula feeding is supported including mastitis, gastroenteritis in the mother or infant, and switching from breast milk to formula (30).

In parallel, Fernandes et al. conducted a 2023 cross-sectional survey in collaboration with the Pediatric European Network for Treatment of AIDS (PENTA) to describe current European infant feeding guidelines (31); expert respondents from 20 countries were selected based upon their involvement in the European AIDS Clinical Society (EACS), and their respective countries' size and burden of HIV (31). In the PENTA survey, 23 respondents from 16 countries reported that half of the countries' guidelines supported breastfeeding for infants born to PLWH (i.e., Switzerland, UK, Germany, Netherlands, Poland, Ireland, Ukraine, Sweden), while the other half either did not support breastfeeding (i.e., Belgium, Denmark, Spain, France, Israel, Latvia, Romania) or did not specify (i.e., Italy) (31). Criteria for supported breastfeeding in the setting of maternal HIV in the countries in former half included two measurements of a VL <50 copies/mL in the third trimester, “good” ART adherence, willingness to undergo more frequent VL testing, and consistent engagement with the healthcare team (31). In the setting of an increase in maternal VL, in addition to cessation of breastfeeding, respondents additionally recommended conducting additional infant HIV testing (Netherlands, Germany, Poland, Ukraine, UK, Ireland) and post-exposure prophylaxis for the infant (Sweden, UK, Ireland) (31).

These surveys demonstrate the heterogeneity of guidelines across Europe. Promoting continued communication and collaboration through organizations like EACS and WAVE in Europe, and the WHO globally, may aid in harmonization of guidelines. Notably, as of 2025 the EACS promotes support for breastfeeding among PLWH who are fully adherent and virally suppressed on continuous ART (32).

Following the 2023 DHHS guideline change to encourage patient-centered counseling inclusive of breastfeeding and shared decision-making around infant feeding, the Canadian Journal of Obstetrics and Gynecology (JOGC) updated their guidelines on care of pregnant PLWH and reducing perinatal transmission (33). Similar to many European national Guidelines, the JOGC guidelines continue to recommend formula feeding regardless of maternal VL or use of ART, the updated guidelines newly emphasized a supportive, non-judgmental approach to care regardless of infant feeding method acknowledging financial and cultural barriers to replacement feeding. The JOGC additionally advises additional parameters to minimize the risk of perinatal infection through breastfeeding, namely that if there is ongoing risk for new HIV infection acquisition, including prioritization of a coordinated care team with appropriate specialists for the mother-infant dyad (33).

### 3.4 Intellectual humility and ethics

Intellectual humility, or the ability to recognize the limitations of one's knowledge while being open to new ideas, is essential not only in the ability to counsel patients and come to a shared decision, but in continuing to revise guidelines on infant feeding as we learn more about the experiences of PLWH, their healthcare providers, and others involved in the decision-making process (34). Acknowledging that the previous guidelines were outdated and/or incomplete may also help build trust between a patient and their provider. Relational decision-making, a model built on this concept of intellectual humility, can be used in infant feeding conversations. It is important to acknowledge and mitigate the power dynamic between a healthcare team and PLWH (34). In a piece on clinical equipoise in the context of HIV and infant feeding, Rudin et al. discuss the uniqueness of HIV in that, unlike other diseases, any residual risk of HIV transmission has been deemed unacceptable by practitioners and governing bodies, leading to hesitation to change guidelines even when they would eliminate other potential risks (35). Rudin et al. believe that the benefits from breastfeeding under optimal conditions outweigh the risk of potential risks, which are very low. Healthcare providers must learn to manage anxiety surrounding very low transmission under optimal conditions, even when faced with an alternative zero-risk strategy (36).

The revised DHHS guidelines are unique in the sense that they were updated not only due to new scientific information, but also to ethical considerations and pressure from advocates (37). Nightingale et al. expand on the ethical considerations of the infant feeding decision from the lens of the four fundamental principles of ethical healthcare: patient autonomy, justice, beneficence, and non-maleficence. They write, “Patient autonomy can only be fully realized when counseling is unbiased, complete, and presented in an accessible manner” (37). They also condemn the criminalization of HIV and the threat of Child Protective Services (CPS) in upholding justice for PLWH. Finally, the concept of clinical equipoise is brought up again in managing beneficence and non-maleficence: “Communication and coordination of care across the mother-child dyad's provider team is also essential to ensure that benefits are achieved while preventing unintended harm.” (37) ANAC called for more of the same in their statement: reformation of policies that criminalize breastfeeding in PLWH and equitable access to HIV-informed lactation support through telehealth (20).

Infant feeding counseling for virally suppressed PLWH is a balancing act between protecting the infant from a very low risk of HIV transmission and respecting the mother's bodily autonomy and decision-making capacity. Best practices include remaining open-minded about the current data and leading with a patient-centered approach.

### 3.5 Provider perspectives

Although health care providers play a critical role in guiding infant feeding decisions for PLWH, there is limited published literature on provider counseling practices (outside of several institutional protocols) or provider perspectives. Early evidence suggests variable uptake and adoption of the updated guidelines by U.S. institutions. A 2023 survey of 100 U.S.-based providers conducted in 2021 by Lai and colleagues found that providers' comfort level with infant feeding counseling and counseling practices for PLWH varied widely. Notably conducted prior to the shift in guidelines, 86% of providers reported counseling PLWH on infant feeding choices, and a significantly smaller subset (56%) of respondents reported counseling specifically about the possibility of breastfeeding (25). The majority of respondents (58%) reported challenges to supporting the decision to breastfeed among PLWH, and multiple factors influenced provider comfort and clinical decision-making in counseling PLWH about breastfeeding (25). Providers cited patient-specific factors such as detectable VL, mental health conditions, low health literacy, and language barriers as barriers to counseling PLWH about breastfeeding, as well as inconsistencies between national guidelines and their institutional guidelines and unresolved concern regarding the risk of transmission to the infant. Across 84 institutions surveyed, only eight institutions had protocols to specifically address breastfeeding among PLWH, prior to the shift in DHHS guidelines (25).

Rozen Eisenberg et al. conducted a cross-sectional study of 130 Pediatric Infectious Disease (PID) Society members, specifically attending physicians and nurse practitioners in the U.S.; 79% of respondents reported that their institution had planned to implement the updated guidelines (38) Importantly, Rozen Eisenberg and colleagues specifically limited their cohort to clinicians who self-reported guideline implementation at their respective institutions, which may capture a higher representation of institutions with established protocols. In contrast, in a cross-sectional study of 397 practicing neonatologists and PID in the U.S. by Ikeri et al., only 35% of PID physicians and 28% of neonatologists reported the centers they practiced at had established guidelines for breastfeeding among PLWH (39). These differences may be partially explained by variations in study design and participant population. These data from Lai et al., Rozen Eisenberg et al., and Ikeri et al. highlight the variability in guideline uptake and implementation of standardized protocol across U.S health care institutions.

Studies across high-income countries demonstrate that providers employ a variety of clinical practices to determine eligibility for breastfeeding, virologic testing schedules for mothers and their infants, and neonatal post-exposure prophylaxis (29, 31, 38, 39). In the U.S., Rozen Eisenberg and colleagues found that infectious disease practitioners demonstrate substantial heterogeneity in their clinical practices when counseling PLWH on breastfeeding safety and eligibility. For example, 34% of respondents reported requiring an undetectable VL across all trimesters for the patient to be considered eligible for breastfeeding and 31% recommended continuation of infant antiretroviral prophylaxis through the end of weaning (38). Similarly, Ikeri and colleagues identified variability in both clinical practices overall and across different specialties, particularly neonatology and pediatric infectious diseases (39). PID specialists (64%) were more likely to recommend breastfeeding from a PLWH with an undetectable VL compared to neonatologists (42%) (39). In both studies, variability in clinical practices may be explained by continued concern for perinatal HIV transmission, insufficient evidence for infant virologic testing frequency, and lack of institutional policies and guidance (38, 39).

Providers also described a spectrum of counseling styles, ranging from prioritizing patient autonomy in the decision to breastfeed adopting more traditional paternalistic approaches (25). Providers who prioritized patient autonomy focused on trust-building, counseling, adherence support, and increased monitoring for PLWH who chose to breastfeed. Providers who cited paternalistic styles when counseling patients described efforts to recommend against breastfeeding for PLWH through reliance on institutional/hospital policies, educating patients regarding the risk of transmission, and rarely threatening CPS involvement (25). Table 3 provides a thematic summary of provider perspectives.

**Table 3:** Thematic summary of provider perspectives on breastfeeding counseling in the setting of maternal HIV (21, 25, 27, 34–36).

Theme	Key findings
Counseling styles	Providers across specialties reported variability in infant feeding counseling approaches, comfort levels, and recommendations due to concern over transmission risk and lack of standardized institutional protocols.
Institutional variability	The uptake of the updated DHHS guidelines and presence of formal protocols varied substantially across institutions.
Clinical practices	Clinicians across and within specialties (e.g., infectious diseases, OB/GYN) employ various and nonuniform clinical practices related to eligibility criteria, infant prophylaxis, and virologic monitoring.
Multidisciplinary support	Coordinated, multidisciplinary care with infectious disease specialists, pediatricians, OB/GYN’s, and lactation specialists is imperative for providing patient-centered, supportive care.

There is a need for additional research on how to more effectively involve lactation support specialists. Barr and colleagues published a call for research on knowledge, attitudes, and practices among lactation support specialists, citing that they are well-positioned to address the intersectional psychosocial, physiological, and emotional aspects of infant feeding decisions for PLWH (40). Additionally, telelactation increases access to quality lactation support and improves outcomes such as higher rates of exclusive breastfeeding at six months postpartum (40). Further investigation of telelactation can be integrated into perinatal care would be invaluable for improving outcomes for PLWH.

As clinical practice shifts to adapt to the updated national guidelines, there is an opportunity for health care providers across multiple disciplines to play a central role in educating and supporting infant feeding choices for PLWH. Discussions about breastfeeding are nuanced, and require navigation of evolving clinical guidelines, ethical considerations, and patient-provider dynamics, while also responding to individual patient needs. There is also a clear need for institutions to provide uniform, evidence-based guidance to health care providers across specialties.

### 3.6 Patient experiences, challenges, and facilitators

Several publications describe how the infant feeding decision-making experience for PLWH is shaped by the interplay of patient knowledge and awareness, personal beliefs, sociocultural influences, and interactions with the healthcare system (41–43). PLWH navigate not only medical risk and safety, but also concerns related to autonomy, HIV disclosure, and stigma. Table 4 summarizes of salient themes from publications describing the patient experience of deciding upon an infant feeding method in the setting of maternal HIV.

**Table 4:** Thematic summary of patient experiences during infant feeding decision-making (11, 12, 37–39, 41–49).

Theme	Key findings
Knowledge and perceptions	Perceptions of breastfeeding safety among PLWH may play a strong role in influencing a patient’s decision-making, though knowledge of safety may be variable.
Motivations for breastfeeding	Mothers highlighted infant bonding and infant health benefits as key motivators for breastfeeding.
Concerns regarding transmission risk	Concern of transmission risk to the infant during breastfeeding and the uncertainty of breastfeeding safety emerged as key factors for a mother's decision to not breastfeed.
Provider communication	Participants highly valued the collaborative, supportive, and informative discussions with their providers.
Stigma and HIV disclosure	Mothers reported a fear of implicit HIV status disclosure and stigma if they chose to not breastfeed, especially in communities where breastfeeding was framed as an expectation.
Sociocultural factors	Mothers frequently noted cultural beliefs, family expectations, and gendered norms related to breastfeeding.

Patient perceptions of breastfeeding are a key determining factor in decision-making. Moseholm and colleagues found that patient perceptions and knowledge of the safety of exclusive breastfeeding while living with HIV played a key role in the decision-making process for infant feeding. In their survey of 44 women living with HIV of Nordic and non-Nordic origin residing in a Nordic country, 75% of participants across both Nordic and non-Nordic groups were aware that it was unsafe to breastfeed with a detectable VL. However, the knowledge of breastfeeding safety with an undetectable VL varied by demographic groups, with several participants reporting they were unsure of whether breastfeeding was safe despite being virally suppressed (43). Participants who had immigrated to a Nordic country from African countries cited difficulty with reconciling differing national guidelines, noting that breastfeeding was recommended in their country of origin, but discouraged in Nordic countries (43).

Several studies describe maternal motivation for breastfeeding (15, 41, 44). A qualitative study of PLWH in Philadelphia by Zapata Vaca and colleagues explored personal motivations and beliefs regarding breastfeeding shape infant feeding choice. Participants frequently cited their perception of breastfeeding as more natural, healthier for the infant, and as an opportunity for bonding with their child as motivation for breastfeeding (41). In a longitudinal study of 8,513 live-birth deliveries from PLWH in the UK from 2012 to 2021, Francis and colleagues similarly found motivations for breastfeeding included bonding with their infant, health benefits of breast milk, and HIV disclosure concerns (15). Participants also cited concerns related to using formula for their infants, including perceived loss of health benefits compared to using breast milk, risk of formula intolerance, and formula shortages (41). Participants who chose not to breastfeed mentioned that their decision to formula feed was influenced by concern for HIV transmission, lack of interest in breastfeeding, convenience of formula feeding, and perceived risk to the infant from maternal medications (41).

Communication and interactions with health care providers is an important factor in how PLWH navigate infant feeding decisions (42, 45–49). A mixed-methods study by Lazenby and colleagues assessed attitudes of breastfeeding among PLWH and their perception of communication regarding infant feeding from their providers. The study found that nearly all participants (99%; *n* = 100) reported trusting their healthcare provider's recommendations on infant feeding (42). Participants frequently reported engaging in shared decision-making with their health care provider regarding infant feeding choice, which allowed healthcare providers to understand the patient's goals and respect their autonomy. Some participants also reported receiving conflicting advice from health care providers regarding the risk of transmission from breastfeeding, which negatively impacted their perceived quality of care and patients' willingness to engage in shared decision-making with their provider (42).

Similarly, in multiple qualitative studies, participants placed high value on the information and support provided by their clinicians, which allowed them to make informed and safe decisions about infant feeding (45, 49, 50). Positive and supportive communication with health care providers allowed PLWH to navigate decision-making and feel empowered in their infant feeding choice. Across these qualitative studies, participants consistently emphasized the importance of clear, supportive, and unbiased interactions that facilitated trust and informed decision-making (45, 49–51). Participants who had access to counseling by a multidisciplinary health care team and coordinated care reported feeling more confident and supported in their decision (49). However, PLWH also described challenges navigating inconsistent messaging across specialties and inadequate access to clear guidance and information regarding breastfeeding (45, 50); in a study by Pagano-Therrien et al., participants frequently reported receiving directive, paternalistic counseling from their clinicians, in which PLWH were explicitly discouraged from breastfeeding, as per prior U.S. guidelines, without the opportunity for further discussion (51). These findings underscore that positive, patient-centered communication and evidence-based guidance is essential for trust-building and autonomy for PLWH.

Sociocultural context also influences how PLWH approach infant feeding; multiple studies highlighted how the risk of HIV disclosure associated with the decision to not breastfeed was a significant factor shaping infant feeding choices, particularly in settings where breastfeeding is socially and culturally expected (15, 41, 42, 49, 50). Zapata Vaca and colleagues write that participants reported experiencing scrutiny and pressure from family and friends to breastfeed, especially those who were not aware of the participant's HIV status (41). Similarly, Levison and colleagues described that in certain ethnic communities, the decision to not breastfeed was perceived as an implicit disclosure of the individual's HIV status (16). Breastfeeding may also be framed as a gendered and cultural expectation for PLWH (49, 50, 52). In a mixed-methods study of PLWH in the Netherlands, one respondent reported, “In my culture, the only thing that makes you a mother is breastfeeding” (52). As a result, PLWH not only weigh medical considerations in their infant feeding decisions, but also the social and cultural consequences of not breastfeeding and concerns about stigma and unintended disclosure.

Family and community involvement further shape patient decision-making for infant feeding choices. Multiple studies demonstrate that influence from family and community members is a notable factor that can both support an also complicate infant feeding decision-making (16, 45–47, 49). In a qualitative study of 36 pregnant PLWH and 2 male partners in the UK, Kasadha and colleagues found that women in relationships with the father of their child valued their partner's support and opinion and participated in joint decision-making (53). However, among participants who were not in a relationship or estranged from their partner, the father was less likely to be involved in the decision-making process due to concerns of intimate partner violence or risk of HIV status disclosure (53). Participants also cited methods to conceal their infant feeding choice from their family members due to the fear of HIV status disclosure (47, 51). Overall, these findings highlight the complex interplay between relationship dynamics, social and cultural expectations, and concerns of HIV status disclosure in an individual's infant feeding decision-making process.

Infant feeding decision-making among PLWH may also have implications for their mental and emotional health, as PLWH are already at a heightened risk of PPD (46). Harris and colleagues evaluated the relationship between infant feeding decision-making and maternal mental health prior to the shift in the DHHS guidelines (2021–2022) within an HIV Prevention and Treatment Services program at Children's National Hospital in Washington, D.C (46). The study found that among 106 participants, nearly 20% of postpartum PLWH experienced PPD 1-month postpartum. A significant proportion of participants who experienced PPD 1 month after delivery reported experiencing “sadness” due to not being able to breastfeed (*n* = 15; 75%). Additionally, more than half of the respondents reported a lack of discussion with their health care provider about the option to breastfeed, suggesting that gaps in counseling and support may contribute to the emotional burden associated with infant feeding decision-making for PLWH.

It is vital to continue investigating the beliefs, attitudes, and experiences of PLWH. Future research regarding the motivations and experiences of PLWH should make strong efforts to include marginalized communities and mitigate research bias (54). Kasadha and colleagues highlight the need to contextualize the lived experiences of PLWH during infant feeding discussions and ensure diverse research teams are involved in investigating these experiences (54). Continued research on patient experiences should inform culturally sensitive counseling practices, reduce stigma of breastfeeding, and support shared decision-making.

For PLWH, infant feeding decision-making is a complex process shaped by access to clear and evidence-based information, sociocultural influence, personal beliefs about breastfeeding, and interactions with the healthcare system. Fear of perinatal HIV transmission is a commonly cited deterrent against breastfeeding among PLWH (16, 50, 51), yet PLWH also frequently cited positive motivators for breastfeeding, including the opportunity to bond with their infant. PLWH also reported challenges in navigating relationships with their family and community due to the risk of HIV disclosure if they chose to not breastfeed. Lastly, access to effective, patient-centered and culturally sensitive counseling across health care specialties and providers may allow PLWH to make informed decisions and feel more supported when navigating these complex considerations.

## 4 Discussion

Since 2023, much has changed in the landscape of infant feeding for PLWH. This review describes the growing body of literature focused on HIV and infant feeding, including institutional protocols for infant feeding counselling and management, additional data on breastfeeding safety, and work describing both the provider and patient experience. The findings of this review can be viewed through the lens of the shared decision-making framework of healthcare (13), and its underlying principles of autonomy, beneficence, and justice (13); as reviewed above, several publications examined infant feeding in light of these principles, as well as the concept of clinical equipoise. Across studies, both provider - and patient-level factors highlight the need for clearer and more consistent communication regarding breastfeeding among PLWH, most notably on a local/institutional level. Providers and patients alike reported navigating uncertainty related to evolving and changing guidelines and noted ethical concerns between minimizing HIV transmission risk and promoting the principles of ethics in healthcare. While many providers expressed willingness to engage in shared decision-making, several studies highlighted gaps and variability in provider comfort and institutional guidance and support, which may limit effective counseling, or in the terms of the shared decision-making model, conference of agency and support for the decision-making process.

Best practices include supportive communication and counseling from healthcare providers during infant feeding discussion; this communication is crucial both for aligning medical recommendations with patient values and autonomy for PLWH. Clinicians are well positioned to support informed decision-making for PLWH, as studies found high levels of patient trust in providers. Infant feeding conversations should be initiated as early as possible, seeking to understand the patient's wishes. The process should be an ongoing exchange of information between the patient and their provider. As a result of this collaborative decision-making process, we are seeing an increase in the amount of patients that decide to breastfeed. This in turn enables clinicians to collect more data on the safety of breastfeeding in PLWH, which will better inform counseling. The decision to breastfeed or not is personal and is influenced by cultural norms, conversations with healthcare providers, and one's own attitudes and beliefs regarding different forms of infant feeding. Patients trust their healthcare providers to counsel them on the most evidence-based recommendations, emphasizing the need for providers to stay informed and free of paternalistic or coercive approaches.

### 4.1 Limitations and strengths

Many studies included in this review had relatively small sample sizes and were largely retrospective or descriptive in nature with short follow-up periods, given the limited timeframe since 2023. Although this review stressed commonalities between institutions, protocols, and experiences, institutional practices varied considerably across settings, as did provider and patient experiences. These limitations accentuate the need for additional prospective research to more fully capture infant feeding counseling and decision-making experiences for both providers and patients. We do, however, feel that the heterogeneity of the protocols of clinical sites demonstrates multiple ways to implement the new U.S. national guidelines. The practices and protocols described in this review should not be interpreted as representing a uniform, standardized model for engaging PLWH in discussions regarding infant feeding practices. Rather, these variations may reflect how institutions in the U.S. are continuing to adapt their practices and standardize their policies in response to evolving clinical evidence surrounding the safety of breastfeeding among virally suppressed PLWH, especially in light of the DHHS guideline update. Our intent was to capture the full spectrum of published perspectives on infant feeding practices and experiences of each party involved, including guidelines, clinical data, ethical considerations, and clinical experiences of providers and PLWH. Lamentably, we were unable to include studies not in English.

Areas for improvement in this field include ensuring clinical practices and institutions are aligned with national guidelines, mitigating bias in the process of counseling patients, and gathering more data on the safety of exclusive breastfeeding under optimal conditions in high-resource settings. Regardless of a mother's ultimate infant feeding decision, continued support should be the cornerstone of care to minimize MTCT and allow for close communication with mothers. Our review identified key evidence gaps, such as the optimal frequency of maternal and infant testing in the postpartum period. There is a clear need for continued efforts and more rigorous data from large-scale studies regarding implementation of the new infant feeding guidelines. Many calls for research can be found throughout our review, such as the specific ways in which lactation support specialists can be used to improve outcomes for PLWH who opt to breastfeed, as well as continuing to explore the knowledge, attitudes, and beliefs of underrepresented voices in research. Work must also continue to decrease the stigma associated with HIV so that individuals with HIV receive the support they need from friends, family, and community.
