# Supplementary material for: Breastfeeding practices and perspectives in the setting of maternal HIV in high-income countries since the shift in the United States national guidelines: a scoping review
Source: Front Reprod Health. 2026 Jun 26;8:1864784. doi: 10.3389/frph.2026.1864784 (PMC13350190; doi:10.3389/frph.2026.1864784)
Supplement: Supplementary file 1 [file Table1.docx]

Supplementary Material

**Supplementary Table 1: Databases (MEDLINE, Embase, CENTRAL, CINAHL Ultimate, and Web of Science) and Strategies**

| Ovid MEDLINE(R) ALL 1946 to August 07, 2025 | | |
| --- | --- | --- |
| **Date Searched** | 8/8/2025 | |
| **Concept** | **Search String** | **Results** |
| **1 HIV** | ((HIV infection* OR  human immunodeficiency virus infection* OR  HIV positive) AND  (mother* OR  pregnant OR  birthing person* OR  birthing people)).ti,ab,kf. OR  ("mothers living with HIV"  "birthing person* living with HIV" OR  "birthing people living with HIV" OR  "women living with HIV").ti,ab,kf. OR  (HIV Infections/ AND  (Pregnant People/ OR  Mothers/)) | 12,248 |
| **2. Formula fed** | (infant formula* OR  baby formula* OR  formula fed OR  formula feeding OR  bottlefeeding OR  bottle feeding OR  bottlefed).ti,ab,kf. OR  Infant Formula/ OR  Bottle Feeding/ | 18,179 |
| **3. Breastfeed** | (breastfeed* OR  breast feed* OR  breastfed OR  breast fed).ti,ab,kf. OR  Breast Feeding/ | 75,146 |
| **4 Outcomes?** | (physician practice* OR  physician knowledge OR  clinical practice* OR  clinical knowledge OR  clinical compentenc* OR  doctor* practice* OR  "provider practice*" OR  "provider* knowledge" OR  (guideline* ADJ2 adhere*) OR  protocol compliance OR  patient experience* OR  patient satisfaction OR  patient preference OR  patient compliance).ti,ab,kf. OR  Practice Patterns, Physicians'/ OR  Attitude of Health Personnel/ OR  Clinical Competence/ OR  Guideline Adherence/ OR  Patient Preference/ OR  Patient Satisfaction/ OR  Physician-Patient Relations/ OR  Patient Compliance/ OR  Patient Acceptance of Health Care/ OR  Health Knowledge, Attitudes, Practice/ | 1,068,104 |
| **Combo** | 1 AND (2 OR 3) AND 4 | 225 |
| **Limits** | N/A |  |
| **Total** |  | 225 |

| Ovid Embase Classic+Embase 1947 to 2025 August 06 | | |
| --- | --- | --- |
| **Date Searched** | 8/8/2025 | |
| **Concept** | **Search String** | **Results** |
| **1 HIV** | ((HIV infection* OR  human immunodeficiency virus infection* OR  HIV positive) AND  (mother* OR  pregnant OR  birthing person* OR  birthing people)).ti,ab,kf. OR  ("mothers living with HIV"  "birthing person* living with HIV" OR  "birthing people living with HIV" OR  "women living with HIV").ti,ab,kf. OR  (Human immunodeficiency virus infection/ AND  (exp Pregnant Person/ OR  Mother/ OR Adolescent Mother/)) | 17,613 |
| **2. Formula fed** | (infant formula* OR  baby formula* OR  formula fed OR  formula feeding OR  bottlefeeding OR  bottle feeding OR  bottlefed).ti,ab,kf. OR  artificial milk/ OR  Bottle Feeding/ | 28,759 |
| **3. Breastfeed** | (breastfeed* OR  breast feed* OR  breastfed OR  breast fed).ti,ab,kf. OR  Breast Feeding/ | 103,582 |
| **4 Outcomes?** | (physician practice* OR  physician knowledge OR  clinical practice* OR  clinical knowledge OR  clinical compentenc* OR  doctor* practice* OR  "provider practice*" OR  "provider* knowledge" OR  (guideline* ADJ2 adhere*) OR  protocol compliance OR  patient experience* OR  patient satisfaction OR  patient preference OR  patient compliance).ti,ab,kf. OR  Clinical Practice/ OR  health personnel attitude/ OR  Clinical Competence/ OR  Protocol Compliance/ OR  Patient Preference/ OR  Patient Satisfaction/ OR  doctor patient relationship/ OR  nurse patient relationship/ OR  Patient Compliance/ OR  Patient Attitude/ OR  attitude to health/ | 1,615,961 |
| **Combo** | 1 AND (2 OR 3) AND 4 | 325 |
| **Limits** | N/A |  |
| **Total** |  | 325 |

| Ovid EBM Reviews - Cochrane Central Register of Controlled Trials July 2025 | | |
| --- | --- | --- |
| **Date Searched** | 8/8/2025 | |
| **Concept** | **Search String** | **Results** |
| **1 HIV** | ((HIV infection* OR  human immunodeficiency virus infection* OR  HIV positive) AND  (mother* OR  pregnant OR  birthing person* OR  birthing people)).ti,ab,kw. OR  ("mothers living with HIV"  "birthing person* living with HIV" OR  "birthing people living with HIV" OR  "women living with HIV").ti,ab,kw. OR  (HIV Infections/ AND  (Pregnant People/ OR  Mothers/)) | 1,439 |
| **2. Formula fed** | (infant formula* OR  baby formula* OR  formula fed OR  formula feeding OR  bottlefeeding OR  bottle feeding OR  bottlefed).ti,ab,kw. OR  Infant Formula/ OR  Bottle Feeding/ | 2,766 |
| **3. Breastfeed** | (breastfeed* OR  breast feed* OR  breastfed OR  breast fed).ti,ab,kw. OR  Breast Feeding/ | 12,329 |
| **4 Outcomes?** | (physician practice* OR  physician knowledge OR  clinical practice* OR  clinical knowledge OR  clinical compentenc* OR  doctor* practice* OR  "provider practice*" OR  "provider* knowledge" OR  (guideline* ADJ2 adhere*) OR  protocol compliance OR  patient experience* OR  patient satisfaction OR  patient preference OR  patient compliance).ti,ab,kw. OR  Practice Patterns, Physicians'/ OR  Attitude of Health Personnel/ OR  Clinical Competence/ OR  Guideline Adherence/ OR  Patient Preference/ OR  Patient Satisfaction/ OR  Physician-Patient Relations/ OR  Patient Compliance/ OR  Patient Acceptance of Health Care/ OR  Health Knowledge, Attitudes, Practice/ | 114,654 |
| **Combo** | 1 AND (2 OR 3) AND 4 | 22 |
| **Limits** | N/A |  |
| **Total** |  | 22 |

| CINAHL Ultimate (EBSCOhost) | | |
| --- | --- | --- |
| **Date Searched** | 8/8/2025 | |
| **Concept** | **Search String** | **Results** |
| **1 HIV** | XB (("HIV infection*" OR  "human immunodeficiency virus infection*" OR  "HIV positive") AND  ("mother*" OR  "pregnant" OR  "birthing person*" OR  "birthing people")) OR  XB ("mothers living with HIV"  "birthing person* living with HIV" OR  "birthing people living with HIV" OR  "women living with HIV") OR  MH ("HIV Infections" AND  "Mothers+") | 5,093 |
| **2. Formula fed** | XB ("infant formula*" OR  "baby formula*" OR  "formula fed" OR  "formula feeding" OR  "bottlefeeding" OR  "bottle feeding" OR  "bottlefed") OR  MH ("Infant Formula" OR  "Bottle Feeding") | 8,316 |
| **3. Breastfeed** | XB ("breastfeed*" OR  "breast feed*" OR  "breastfed" OR  "breast fed") OR  MH ("Breast Feeding+") | 40,662 |
| **4 Outcomes** | XB ("physician practice*" OR  "physician knowledge" OR  "clinical practice*" OR  "clinical knowledge" OR  "clinical compentenc*" OR  "doctor* practice*" OR  "provider practice*" OR  "provider* knowledge" OR  ("guideline*" N2 "adhere*") OR  "protocol compliance" OR  "patient experience*" OR  "patient satisfaction" OR  "patient preference" OR  "patient compliance") OR  MH ("Practice Patterns" OR  "Professional Practice, Evidence-Based+" OR  "Attitude of Health Personnel" OR  "Clinical Competence" OR  "Guideline Adherence" OR  "Patient Preference" OR  "Patient Satisfaction" OR  "Physician-Patient Relations" OR  "Nurse-Patient Relations" OR  "Patient Compliance" OR  "Attitude to Health") | 536,364 |
| **Combo** | 1 AND (2 OR 3) AND 4 | 84 |
| **Limits** | N/A |  |
| **Total** |  | 84 |

| Web of Science Core Collection: All Editions | | |
| --- | --- | --- |
| **Date Searched** | 8/8/2025 | |
| **Concept** | **Search String** | **Results** |
| **1 HIV** | TS=(("HIV infection*" OR  "human immunodeficiency virus infection*" OR  "HIV positive") AND  ("mother*" OR  "pregnant" OR  "birthing person*" OR  "birthing people")) OR  TS=("mothers living with HIV"  "birthing person* living with HIV" OR  "birthing people living with HIV" OR  "women living with HIV") | 10,538 |
| **2. Formula fed** | TS=("infant formula*" OR  "baby formula*" OR  "formula fed" OR  "formula feeding" OR  "bottlefeeding" OR  "bottle feeding" OR  "bottlefed") | 16,457 |
| **3. Breastfeed** | TS=("breastfeed*" OR  "breast feed*" OR  "breastfed" OR  "breast fed") | 56,838 |
| **4 Outcomes?** | TS=("physician practice*" OR  "physician knowledge" OR  "clinical practice*" OR  "clinical knowledge" OR  "clinical compentenc*" OR  "doctor* practice*" OR  "provider practice*" OR  "provider* knowledge" OR  ("guideline*" N2 "adhere*") OR  "protocol compliance" OR  "patient experience*" OR  "patient satisfaction" OR  "patient preference" OR  "patient compliance") | 438,881 |
| **Combo** | #1 AND (#2 OR #3) AND #4 | 10 |
| **Limits** | N/A |  |
| **Total** |  | 10 |

<><><><><><><><><><><><><><><><>Search Update<><><><><><><><><><><><><><><><>

| Ovid MEDLINE(R) ALL 1946 to January 05, 2026 | | |
| --- | --- | --- |
| **Date Searched** | 1/6/2026 | |
| **Concept** | **Search String** | **Results** |
| **1 HIV** | ((HIV infection* OR  human immunodeficiency virus infection* OR  HIV positive) AND  (mother* OR  pregnant OR  birthing person* OR  birthing people)).ti,ab,kf. OR  ("mothers living with HIV"  "birthing person* living with HIV" OR  "birthing people living with HIV" OR  "women living with HIV").ti,ab,kf. OR  (HIV Infections/ AND  (Pregnant People/ OR  Mothers/)) | 12,493 |
| **2. Formula fed** | (infant formula* OR  baby formula* OR  formula fed OR  formula feeding OR  bottlefeeding OR  bottle feeding OR  bottlefed).ti,ab,kf. OR  Infant Formula/ OR  Bottle Feeding/ | 18,506 |
| **3. Breastfeed** | (breastfeed* OR  breast feed* OR  breastfed OR  breast fed).ti,ab,kf. OR  Breast Feeding/ | 76,601 |
| **4 Outcomes?** | (physician practice* OR  physician knowledge OR  clinical practice* OR  clinical knowledge OR  clinical compentenc* OR  doctor* practice* OR  "provider practice*" OR  "provider* knowledge" OR  (guideline* ADJ2 adhere*) OR  protocol compliance OR  patient experience* OR  patient satisfaction OR  patient preference OR  patient compliance).ti,ab,kf. OR  Practice Patterns, Physicians'/ OR  Attitude of Health Personnel/ OR  Clinical Competence/ OR  Guideline Adherence/ OR  Patient Preference/ OR  Patient Satisfaction/ OR  Physician-Patient Relations/ OR  Patient Compliance/ OR  Patient Acceptance of Health Care/ OR  Health Knowledge, Attitudes, Practice/ | 1,097,724 |
| **5. Combo** | 1 AND (2 OR 3) AND 4 | 234 |
| **6. Limits** | Limit 5 to dt=20250807-20271231 |  |
| **Total** |  | 8 |

| Ovid Embase Classic+Embase 1947 to 2026 January 05 | | |
| --- | --- | --- |
| **Date Searched** | 1/6/2026 | |
| **Concept** | **Search String** | **Results** |
| **1 HIV** | ((HIV infection* OR  human immunodeficiency virus infection* OR  HIV positive) AND  (mother* OR  pregnant OR  birthing person* OR  birthing people)).ti,ab,kf. OR  ("mothers living with HIV"  "birthing person* living with HIV" OR  "birthing people living with HIV" OR  "women living with HIV").ti,ab,kf. OR  (Human immunodeficiency virus infection/ AND  (exp Pregnant Person/ OR  Mother/ OR Adolescent Mother/)) | 18,094 |
| **2. Formula fed** | (infant formula* OR  baby formula* OR  formula fed OR  formula feeding OR  bottlefeeding OR  bottle feeding OR  bottlefed).ti,ab,kf. OR  artificial milk/ OR  Bottle Feeding/ | 29,272 |
| **3. Breastfeed** | (breastfeed* OR  breast feed* OR  breastfed OR  breast fed).ti,ab,kf. OR  Breast Feeding/ | 106,310 |
| **4 Outcomes?** | (physician practice* OR  physician knowledge OR  clinical practice* OR  clinical knowledge OR  clinical compentenc* OR  doctor* practice* OR  "provider practice*" OR  "provider* knowledge" OR  (guideline* ADJ2 adhere*) OR  protocol compliance OR  patient experience* OR  patient satisfaction OR  patient preference OR  patient compliance).ti,ab,kf. OR  Clinical Practice/ OR  health personnel attitude/ OR  Clinical Competence/ OR  Protocol Compliance/ OR  Patient Preference/ OR  Patient Satisfaction/ OR  doctor patient relationship/ OR  nurse patient relationship/ OR  Patient Compliance/ OR  Patient Attitude/ OR  attitude to health/ | 1,670,851 |
| **5. Combo** | 1 AND (2 OR 3) AND 4 | 339 |
| **6 Limits** | Limit 5 to dc=20250807-20271231 |  |
| **Total** |  | 17 |

| Ovid EBM Reviews - Cochrane Central Register of Controlled Trials November 2025 | | |
| --- | --- | --- |
| **Date Searched** | 1/6/2026 | |
| **Concept** | **Search String** | **Results** |
| **1 HIV** | ((HIV infection* OR  human immunodeficiency virus infection* OR  HIV positive) AND  (mother* OR  pregnant OR  birthing person* OR  birthing people)).ti,ab,kw. OR  ("mothers living with HIV"  "birthing person* living with HIV" OR  "birthing people living with HIV" OR  "women living with HIV").ti,ab,kw. OR  (HIV Infections/ AND  (Pregnant People/ OR  Mothers/)) | 1,464 |
| **2. Formula fed** | (infant formula* OR  baby formula* OR  formula fed OR  formula feeding OR  bottlefeeding OR  bottle feeding OR  bottlefed).ti,ab,kw. OR  Infant Formula/ OR  Bottle Feeding/ | 2,805 |
| **3. Breastfeed** | (breastfeed* OR  breast feed* OR  breastfed OR  breast fed).ti,ab,kw. OR  Breast Feeding/ | 12,716 |
| **4 Outcomes?** | (physician practice* OR  physician knowledge OR  clinical practice* OR  clinical knowledge OR  clinical compentenc* OR  doctor* practice* OR  "provider practice*" OR  "provider* knowledge" OR  (guideline* ADJ2 adhere*) OR  protocol compliance OR  patient experience* OR  patient satisfaction OR  patient preference OR  patient compliance).ti,ab,kw. OR  Practice Patterns, Physicians'/ OR  Attitude of Health Personnel/ OR  Clinical Competence/ OR  Guideline Adherence/ OR  Patient Preference/ OR  Patient Satisfaction/ OR  Physician-Patient Relations/ OR  Patient Compliance/ OR  Patient Acceptance of Health Care/ OR  Health Knowledge, Attitudes, Practice/ | 118,118 |
| **Combo** | 1 AND (2 OR 3) AND 4 | 22 |
| **Limits** | 2025-Current |  |
| **Total** |  |  |

| CINAHL Ultimate (EBSCOhost) | | |
| --- | --- | --- |
| **Date Searched** | 1/6/2026 | |
| **Concept** | **Search String** | **Results** |
| **1 HIV** | XB (("HIV infection*" OR  "human immunodeficiency virus infection*" OR  "HIV positive") AND  ("mother*" OR  "pregnant" OR  "birthing person*" OR  "birthing people")) OR  XB ("mothers living with HIV"  "birthing person* living with HIV" OR  "birthing people living with HIV" OR  "women living with HIV") OR  MH ("HIV Infections" AND  "Mothers+") | 5,047 |
| **2. Formula fed** | XB ("infant formula*" OR  "baby formula*" OR  "formula fed" OR  "formula feeding" OR  "bottlefeeding" OR  "bottle feeding" OR  "bottlefed") OR  MH ("Infant Formula" OR  "Bottle Feeding") | 8,439 |
| **3. Breastfeed** | XB ("breastfeed*" OR  "breast feed*" OR  "breastfed" OR  "breast fed") OR  MH ("Breast Feeding+") | 41,203 |
| **4 Outcomes** | XB ("physician practice*" OR  "physician knowledge" OR  "clinical practice*" OR  "clinical knowledge" OR  "clinical compentenc*" OR  "doctor* practice*" OR  "provider practice*" OR  "provider* knowledge" OR  ("guideline*" N2 "adhere*") OR  "protocol compliance" OR  "patient experience*" OR  "patient satisfaction" OR  "patient preference" OR  "patient compliance") OR  MH ("Practice Patterns" OR  "Professional Practice, Evidence-Based+" OR  "Attitude of Health Personnel" OR  "Clinical Competence" OR  "Guideline Adherence" OR  "Patient Preference" OR  "Patient Satisfaction" OR  "Physician-Patient Relations" OR  "Nurse-Patient Relations" OR  "Patient Compliance" OR  "Attitude to Health") | 551,264 |
| **Combo** | 1 AND (2 OR 3) AND 4 | 84 |
| **Limits** | 08/07/2025 to 12/31/2026 |  |
| **Total** |  | 1 |

| Web of Science Core Collection: All Editions | | |
| --- | --- | --- |
| **Date Searched** | 1/6/2026 | |
| **Concept** | **Search String** | **Results** |
| **1 HIV** | TS=(("HIV infection*" OR  "human immunodeficiency virus infection*" OR  "HIV positive") AND  ("mother*" OR  "pregnant" OR  "birthing person*" OR  "birthing people")) OR  TS=("mothers living with HIV"  "birthing person* living with HIV" OR  "birthing people living with HIV" OR  "women living with HIV") | 10,713 |
| **2. Formula fed** | TS=("infant formula*" OR  "baby formula*" OR  "formula fed" OR  "formula feeding" OR  "bottlefeeding" OR  "bottle feeding" OR  "bottlefed") | 16,786 |
| **3. Breastfeed** | TS=("breastfeed*" OR  "breast feed*" OR  "breastfed" OR  "breast fed") | 57,887 |
| **4 Outcomes?** | TS=("physician practice*" OR  "physician knowledge" OR  "clinical practice*" OR  "clinical knowledge" OR  "clinical compentenc*" OR  "doctor* practice*" OR  "provider practice*" OR  "provider* knowledge" OR  ("guideline*" N2 "adhere*") OR  "protocol compliance" OR  "patient experience*" OR  "patient satisfaction" OR  "patient preference" OR  "patient compliance") | 456,291 |
| **Combo** | #1 AND (#2 OR #3) AND #4 | 11 |
| **Limits** | AND DOP=2025-08-07/2027-12-31 |  |
| **Total** |  | 1 |

<><><><><><><><><><><><><><><> Supplemental Search <><><><><><><><><><><><><><><>

| Ovid MEDLINE(R) ALL 1946 to February 03, 2026 | | |
| --- | --- | --- |
| **Date Searched** | 2/4/2026 | |
| **Concept** | **Search String** | **Results** |
| **1 HIV** | (HIV Infections/ AND  (Female/ OR  Pregnancy/)) OR  (parent ADJ2 HIV).ti,ab. | 118,804 |
| **2. Formula fed** | (infant formula* OR  baby formula* OR  formula fed OR  formula feeding OR  bottlefeeding OR  bottle feeding OR  bottlefed).ti,ab,kf. OR  Infant Formula/ OR  Bottle Feeding/ | 18,564 |
| **3. Breastfeed** | (breastfeed* OR  breast feed* OR  breastfed OR  breast fed).ti,ab,kf. OR  Breast Feeding/ | 76,854 |
| **4 Outcomes?** | (guideline* ADJ8  (practice OR  recommend* OR  implement* OR  support*)).ti,ab,kf. OR  ((family centered OR  person centered)  AND  (guidance OR  recommend*)).ti,ab. OR  (parental choice OR  decision making).ti,ab. OR  Decision Making/ OR  Guidelines as Topic/ OR  Practice Guidelines as Topic/ OR  (Societies, Medical/ AND Health Policy/) | 608,705 |
| **5. Combo** | 1 AND (2 OR 3) AND 4 | 314 |
| **6. Limits** | N/A |  |
| **Total** |  | 314 |

**
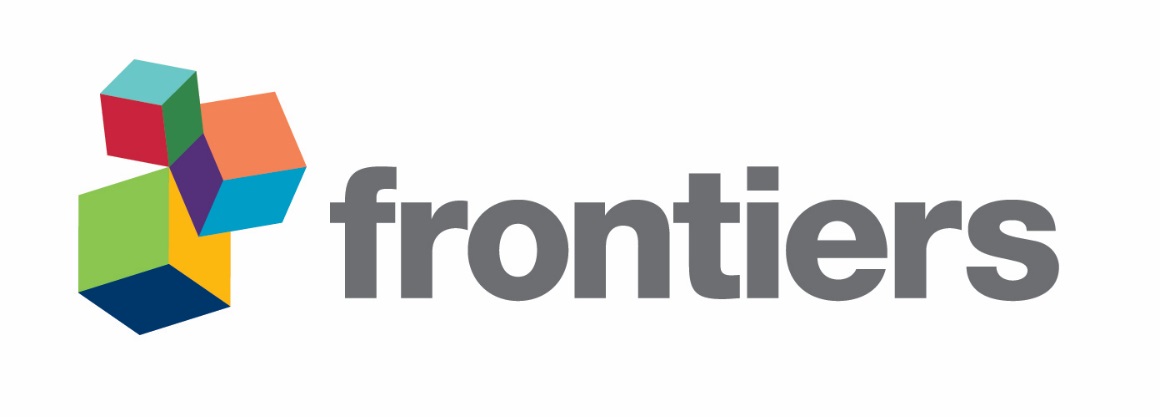
**
