# Supplementary material for: Breastfeeding practices and perspectives in the setting of maternal HIV in high-income countries since the shift in the United States national guidelines: a scoping review
Source: Front Reprod Health. 2026 Jun 26;8:1864784. doi: 10.3389/frph.2026.1864784 (PMC13350190; doi:10.3389/frph.2026.1864784)
Supplement: Supplementary file 2 [file Table2.docx]

| **Supplementary Table 2. Summary table of included sources** | | | | | |
| --- | --- | --- | --- | --- | --- |
| **Study Name** | **First Author**  **Country Publication date** | **Study Aim** | **Population Sample size** | **Study Design** | **Key findings** |
| [*Development and Implementation of an Interdisciplinary Model for the Management of Breastfeeding in Women with HIV in the United States: Experience from the Children’s Hospital Colorado Immunodeficiency Program*](https://www.zotero.org/google-docs/?O1V7HP) | Abuogi  U.S.  2023 | To convene an interdisciplinary group of providers to establish a protocol intended to minimize the risk of vertical transmission during breastfeeding | Cisgender females who breastfed or considered breastfeeding between 2015 and 2022  N=21 women living with HIV who were counseled regarding breastfeeding, of whom, 10 proceeded to breastfeed a total of 13 infants | Cross-sectional | The Children’s Hospital Colorado Immunodeficiency Program observed earlier cessation of breastfeeding in PLWH compared to the general population. The authors postulate that earlier support from the care team may enable patients to breastfeed for prolonged periods. Patients also face challenges such as mastitis, low milk supply, viral blips, and difficulty weaning. |
| [*Infant feeding for persons living with and at risk for HIV in the United States: clinical report.*](https://www.zotero.org/google-docs/?O1V7HP) | Abuogi  U.S.  2024 | To present clinical guidelines and recommendations for pediatric health care professionals regarding infant feeding practices when caring for infants born to PLWH and at risk for HIV infection. | N/A | Clinical Practice Guideline | Refraining from breastfeeding may compound an already elevated risk of poor health outcomes in Black PLWH. |
| *Guideline No. 450: Care of Pregnant Women Living with HIV and Interventions to Reduce Perinatal Transmission* | Atkinson  Canada  2024 | To provide an update on the care of pregnant women living with HIV and the prevention of perinatal HIV transmission. | N/A | Clinical Practice Guideline | Updated Journal of Obstetrics and Gynecology guidelines regarding infant feeding in PLWH. An approach similar to the updated DHHS guidelines is described: a shared decision-making approach with involvement of a multidisciplinary healthcare team. |
| *Infant feeding: emerging concepts to prevent HIV transmission* | Bamford  UK  2024 | To summarize guidelines in countries with various income levels, their differences, and examine the ongoing studies related to vertical HIV transmission | N/A | Review | The authors review guidance from high-income countries regarding breastfeeding among PLWH. The authors describe the evidence demonstrating the low risk of vertical transmission of HIV while breastfeeding if the mother is virally suppressed. The authors also review the literature regarding long-acting ART for maternal preexposure prophylaxis or treatment, as well as infant postnatal prophylaxis. |
| [*A review of updated guidelines on breastfeeding with human immunodeficiency virus using relational decision-making and intellectual humility to support infant feeding choices.*](https://www.zotero.org/google-docs/?O1V7HP) | Barr  U.S.  2024 | To review key infant feeding guidelines updates  published by HHS, as well as  to describe relational decision-making practices that may benefit health care providers when discussing infant feeding. | N/A | Review | The authors discuss the importance of intellectual humility when counseling PLWH on infant feeding. Acknowledging that the previous guidelines were incorrect and/or incomplete may help build trust between a patient and their provider. Relational decision-making can be used in infant feeding conversations. It is important to acknowledge and mitigate the power dynamic between a healthcare team and PLWH. |
| [*Association of nurses in AIDS care position on breastfeeding and chestfeeding: ensuring equity, autonomy, access, and respect in HIV-related infant feeding decisions*](https://www.zotero.org/google-docs/?O1V7HP) | Barr  U.S.  2025 | To affirm the importance of access, equity, autonomy, and respect in lactation counseling and care. The statement includes recommendations for education, policy reform, and implementation research to improve care deliver for PLWH. | N/A | Expert Opinion | Statement in 2025 in support of person-centered counseling with an emphasis on relational decision-making, continuity of care, and trauma-informed practices. |
| [*Lactation support for breastfeeding and chestfeeding people with HIV: a call for research to examine telelactation experience, HIV knowledge, and lactation consultants’ attitudes related to infant feeding with HIV.*](https://www.zotero.org/google-docs/?O1V7HP) | Barr  U.S.  2025 | To call for more research to identify possible gaps in knowledge and other needs within the lactation support community so that infant feeding specialists are adequately equipped with evidence-based strategies to support the unique needs of parents living with HIV. | N/A | Expert Opinion | The authors call for more research on the role of lactation consultants and telelactation to improve breastfeeding for PLWH. The authors highlight the supportive role of lactation specialists and telelactation services to increase access to lactation support. |
| *Updates in the Management of HIV During Pregnancy* | Benson  U.S.  2024 | To summarize and provide updated recommendations for the care of PLWH during pregnancy. | N/A | Review | In parents who are breastfeeding, ART adherence support and monitoring of maternal VL remains paramount as ART adherence (and, by extension, maternal VL) are not infrequently impacted by postpartum depression, in addition to the responsibilities of childcare and an altered sleep schedule. |
| *From Guidelines to Practice: A Programmatic Model for Implementation of the Updated Infant Feeding Recommendations for People Living with HIV* | Boyce  U.S.  2024 | To describe the successful approach utilized in Wisconsin, as a blueprint for other centers to operationalize the DHHS  guidelines. | Postpartum PLWH  N=178 | Retrospective chart review | HIV infection was excluded in 5 out of 7 breastfed infants. One infant was lost to follow-up, but upon re-establishment of care, tested positive for HIV. The maternal viral load was also high, suggesting nonadherence to ART. Also contains information for updated guideline implementation. |
| *Opportunities and Constraints to Equitable Implementation of the Revised Infant Feeding Guidelines for Pregnant and Lactating Persons Living With HIV in the United States: A Qualitative Study* | Buckland  U.S.  2025 | To identify opportunities and constraints to equitable adoption and implementation of the revised infant feeding guidelines for pregnant and lactating PLWH in the U.S. | Service providers  N=15  Pregnant/  lactating PLWH  N=7 | Qualitative | Both providers and patients in this study were largely supportive of the guideline shift due to improved patient autonomy and destigmatization of breastfeeding for PLWH. Participants also discussed several barriers to implementing these guidelines due to concern for risk of transmission, inconsistencies in provider education, and structural barriers such as fragmented, siloed care. |
| *Exploring the Breastfeeding Desires and Decision-Making of Women Living with HIV in the Netherlands: Implications for Perinatal HIV Management in Developed Countries* | Bukkems  Netherlands2023 | To explore breastfeeding desires and decision-making of  immigrant and nonimmigrant women living with HIV in the Netherlands. | Women living with HIV in the Netherlands  N=82 | Cross-sectional | More than 70% of participants expressed a desire to breastfeed in the future, and a majority stated a willingness to undergo additional monitoring if breastfeeding. |
| *Breastfeeding in women with HIV infection: A qualitative study of barriers and facilitators* | Chaparro  U.S.  2024 | To identify factors influencing infant feeding choices decisions among PLWH in a multiethnic and multicultural population. | Women living with HIV (who had given birth within 6 months  N=20 | Qualitative | The authors identified several barriers to breastfeeding including the fear of transmission, lack of standardized education, and logistical/practical concerns. They also identified multiple facilitators: perceived health benefits of breastmilk, infant bonding, and supporting patient autonomy. |
| *Parenting with HIV: a patient's view on updated infant feeding guidelines in the US* | Covin  U.S.  2024 | Personal narrative describing a parent’s perspective on the guideline shift | N/A | Expert Opinion | The author describes their experience as a Black PLWH and their advocacy for breastfeeding. The author describes challenges with medical providers during discussions regarding infant feeding. The author emphasizes the need for education for providers and patients in light of the updated guidelines. |
| *Successful implementation of new Swiss recommendations on breastfeeding of infants born to women living with HIV* | Crisinel  Switzerland  2023 | To describe the motivational factors and the outcome of women with HIV who breastfeed, and of their infants. | Pregnant women living with HIV in Switzerland  N=41 | Nested observational | Among the 41 participants who gave birth, 25 chose to breastfeed. There was no evidence of HIV transmission to the infant at least 3 months after weaning. The main motivations for breastfeeding among this cohort included perceived maternal and infant health benefits, and bonding with the infant. |
| *Advances in HIV Management During Pregnancy and Infant Feeding* | Espinal  U.S.  2024 | To review the latest advancements in HIV management that are revolutionizing pregnancy and postpartum care in pursuit of elimination of perinatal HIV  transmission. | N/A | Systematic review | Although adherence to ART and sustained viral suppression during pregnancy and  breastfeeding reduce breast milk-associated HIV transmission to less than 1%, HIV viral  reservoirs can remain present in breast milk despite an undetectable plasma viral load. Formula or pasteurized donor human milk are the only infant feeding options that  completely eliminate the risk of postnatal HIV transmission. |
| *Breastfeeding in HIV-positive mothers under optimized conditions: 'real-life' results from a well-resourced healthcare setting* | [Feiterna-Sperling](https://www.zotero.org/google-docs/?O1V7HP)  Germany  2025 | To evaluate mother to child transmission of HIV during breastfeeding, as well as breastfeeding practices (exclusive vs. mixed) among mothers living with HIV under optimized conditions in a resource-rich setting | Women living with HIV who gave birth to infants and seen at the Charité – Universitätsmedizin Berlin  N=77 | Longitudinal cross-sectional | The authors noted an increasing proportion of PLWH who opted to breastfeed each year from 2017-2023. HIV infection was excluded in 75/77 infants at the time of publication. |
| *HIV postnatal prophylaxis and infant feeding policies vary across Europe: results of a Penta survey* | Fernandes  Europe  2025 | To describe current European postnatal  prophylaxis and infant feeding policies with the aim of informing future  harmonized guidelines. | Twenty countries in Europe  N=23 pediatricians | Cross-sectional | Eight out of 16 countries surveyed had guidelines that recommended against breastfeeding in the setting of maternal HIV, and the other half supported breastfeeding if certain criteria were met, such as good ART adherence, two measurements of viral load <50 copies/mL, and consistent engagement with the healthcare team. Guidelines for managing viral blips are also discussed. |
| *Monitoring clinical practice of BHIVA-supported breastfeeding guidelines for women living with HIV in the UK* | [Francis](https://www.zotero.org/google-docs/?O1V7HP)  UK  2023 | To describe clinical practice of supported breastfeeding using population-level data from 2012-2021. | All pregnancies to diagnosed women living with HIV in the UK  N=8,513 live births | Longitudinal cross-sectional | The proportion of PLWH opting to breastfeed increased four-fold between 2012 and 2021, likely due to more patient-centered counseling practices. HIV infection was excluded in 150 out of 203 infants at the time of publication. |
| *The Mental Health Effects and Experiences of Breastfeeding Decision-Making Among Postpartum Women Living with HIV* | Harris  U.S.  2024 | To screen PLWH for postpartum depression, evaluate their attitudes toward breastfeeding, and assess their experience with breastfeeding decision-making. | PLWH who presented  for HIV testing of their infants.  N=106 | Cohort | Over 37% of participants reported feelings of “sadness” associated with being unable to breastfeed. The majority of participants (60%) reported not having a discussion with their provider regarding infant feeding, and only half of participants were aware of the guideline shift. The authors highlight the need for providing mental health support and improving information sharing from providers for PLWH. |
| *Adoption of the Revised DHHS Guidelines on Breastmilk Feeding and HIV in the United States: Clinical Practices and Barriers* | Ikeri  U.S.  2025 | To assess the proportion of sub-  specialists who do not recommend breastmilk as a feeding option. The authors also evaluated the extent of national adoption of the updated infant feeding guideline and identified barriers to implementation among subspecialists directly involved in infant feeding decision-making in the U.S. | 389; Actively practicing neonatologists  and pediatric infectious diseases physicians in the U.S. | Cross-sectional | Pediatric infectious disease specialists were more likely than neonatologists to support breastfeeding among virally suppressed PLWH. The strongest barrier to supporting breastfeeding was concern for HIV transmission, among both neonatologists and pediatric infectious disease specialists. |
| *Exploring the complexities of infant feeding decisions for immigrant pregnant people living with HIV in Ontario, Canada: a qualitative study* | John  Canada  2026 | To explore the lived experiences of PLWH in making decisions about infant feeding to better prepare healthcare providers for patient discussions and to enhance guidelines that consider breast milk use among infants born to PLWH | PLWH who delivered between 2018 and 2023 at a referral hospital in Toronto; all participants were immigrants.  N=10 | Qualitative | The authors highlight the opposing recommendations from North American societies to formula feed, and from the World Health Organization to exclusively breastfeed. The authors emphasize the need for multidisciplinary, culturally-sensitive counseling and access to information for PLWH when engaging in discussions regarding infant feeding, especially for PLWH who have immigrated. |
| [*How women living with HIV in the UK manage infant-feeding decisions and vertical transmission risk - a qualitative study.*](https://www.zotero.org/google-docs/?O1V7HP) | Kasadha  UK  2024 | To investigate infant-feeding decision-making among women  living with HIV. | UK-based  individuals with a confirmed HIV diagnosis who were pregnant or one-year postpartum  N=36 | Qualitative | Infant feeding decision-making was largely influenced by access to information, social implications of infant feeding choice, and logistical/practical concerns. The authors highlight the need for clinicians to consider the personal, social and financial concerns of PLWH when engaging in discussions regarding infant feeding. |
| [*“We decided together”: a qualitative study about women with HIV navigating infant-feeding decisions with the father of their children.*](https://www.zotero.org/google-docs/?O1V7HP) | Kasadha  UK  2024 | To explore how fathers influence infant-feeding decisions in the context of preventing vertical transmission of HIV. | UK-based  pregnant postpartum PLWH  N=36 | Qualitative | PLWH who reported being in a relationship described valuing the support and input from the father of their child during infant feeding decision-making. PLWH who were not in a relationship were less likely to discuss their decision with the father, with some participants citing safety concerns and fear of HIV disclosure. |
| [*Hearing the silence and silenced: co-producing research on infant-feeding experiences and practices with Black women with HIV.*](https://www.zotero.org/google-docs/?O1V7HP) | Kasadha  UK  2025 | To discuss experiences of co‐producing research on infant‐  feeding experiences and practices among women with HIV | N/A | Qualitative | The authors discuss the importance of inclusive, diverse research teams when producing research on infant feeding practices among PLWH. In particular, the authors highlight the need to include racially diverse individuals and PLWH within the study teams. |
| [*Guidelines and practice of breastfeeding in women living with HIV-results from the European INSURE survey.*](https://www.zotero.org/google-docs/?O1V7HP) | Keane  Europe  2024 | To collate  information on breastfeeding trends, practice, and guideline recommendations  for women living with HIV in Europe and to establish interest in a collaborative network. | Topical experts across Europe.  N=25 | Cross-sectional | 12 out of 23 countries’ guidelines recommended against breastfeeding, while 11 supported breastfeeding under certain criteria. None of them offered an option for all women to breastfeed. |
| [*European guidelines on HIV and breastfeeding: “Same, same, but different” - results from a WAVE survey*](https://www.zotero.org/google-docs/?O1V7HP) | Keane  Europe  2025 | To consolidate European guidelines in order to better inform medical staff and PLWH in the infant feeding decision-making. | Topical experts representing 20 countries across Europe.  N= 23 representatives of 20 countries | Qualitative | Seven out of 20 countries surveyed had guidelines against breastfeeding. Twelve support breastfeeding under certain criteria. The guidelines are further delineated by viral load monitoring and the ways they address potential challenges faced by patients. |
| *Tilting the Scale: Current Provider Perspectives and Practices on Breastfeeding with HIV in the United States* | Lai  U.S.  2023 | To describe how providers nationally navigate infant feeding with PLHIV. This study utilizes a mixed methods national survey to describe U.S.  medical provider practice, comfort, and perceived challenges relating to breastfeeding in the setting of maternal HIV. | U.S. based providers with experience in breastfeeding in the setting of maternal HIV  N= 100 respondents from 84 institutions | Mixed-methods | The authors found that providers balance discomfort regarding the risk of concern of transmission to the infant, while also supporting patient autonomy in infant feeding decisions. Additionally, the reported lack of institutional guidelines was associated with lower comfort levels in recommending breastfeeding to patients in clinical vignette scenarios. Only 10% of 84 institutions identified by participants have an institutional policy regarding breastfeeding among PLWH. |
| *Attitudes on breast feeding among persons with HIV who have given birth and their perceptions of coercion during counseling on safe infant feeding practices* | Lazenby  U.S.  2023 | To assess if PLWH felt coerced to formula feed when counseled about practices to reduce HIV transmission | PLWH who had delivered a viable infant  N=100 | Mixed-methods | The authors suggest that participants generally held positive views towards breastfeeding; only a small subsection of participants (n = 13) reported coercion to breastfeed. |
| *Breastfeeding Among People With Human Immunodeficiency Virus in North America: A Multisite Study* | Levison  U.S. & Canada  2023 | To characterize people who breastfeed, including their motivations, challenges, and facilitators, and duration of breastfeeding, and to describe institutional practices surrounding breastfeeding for patients with HIV, including counseling, infant prophylaxis, and infant and maternal monitoring. | Individuals with HIV who breastfed during 2014–2022 in the U.S. (8 sites) and Canada (3 sites)  N=72 patients at 11 sites | Retrospective multi-site | Disclosure of HIV status to either partner or friends/family can aid in breastfeeding. Challenges of breastfeeding include low milk supply, pain, mastitis, cracked nipples, and difficulty latching. HIV was excluded in 68/72 infants. |
| *Providers Have a Responsibility to Discuss Options for Infant Feeding With Pregnant People With Human Immunodeficiency Virus in High-Income Countries* | McKinney  U.S.  2023 | To review the overall state of evidence on infant-feeding practices for PWLH, point out the gaps in the current infant-feeding guidelines in the U.S., and describe one approach to counseling and supporting pregnant PLWH who  choose to breastfeed their infant. | N/A | Review | This study represents a framework for guideline implementation. Unique to their method is a signed contract between physician and patient that is meant to be a show of support for their decision. |
| *Infant feeding knowledge among women living with HIV and their interaction with healthcare providers in a high-income setting: a longitudinal mixed methods study* | Moseholm  Denmark, Finland, Sweden  2024 | To understand infant feeding knowledge among women living with HIV of Nordic and non-Nordic origin living in Nordic countries, and their interaction with healthcare providers regarding infant feeding planning. | Pregnant women living with HIV  Survey: N=44, Interviews: N=31 | Mixed-methods | Both Nordic and non-Nordic participants expressed confusion regarding the safety of breastfeeding while virally suppressed. Participants highlighted concerns for increased infant testing and infant ART exposure. Many participants described the need for more education regarding the safety of breastfeeding. |
| *When Black and White Turns Gray: Navigating the Ethical Challenges of Implementing Shared Infant Feeding Decisions for Persons Living with Human Immunodeficiency Virus in the United States* | Nightingale  U.S.  2024 | To review the complex ethical considerations applicable to implementation of the new guidelines as they relate to autonomy, justice, and beneficence. | N/A | Review | The authors expand upon the 2023 DHHS guidelines. The authors highlight how influence from advocates, ethical considerations of patient autonomy, and emerging evidence on breastfeeding safety led to the guideline shift. The authors discuss the ethics of infant feeding in PLWH through the lens of the 4 tenets of medicine: beneficence, non-maleficence, autonomy, and justice. |
| *"Go With the Flow": A Qualitative Description of Infant Feeding Experiences Among Women With HIV in the United States* | Pagano-Therrien  U.S.  2023 | To describe the experiences, beliefs, and feelings of women with HIV in the U.S. surrounding recommendations for breastfeeding avoidance. | PLWH with pregnancy or birth of child within 5  years  N=8 | Qualitative | There is a strong necessity of high-quality lactation care and support for PLWH. The authors recommend respectful, patient-centered care that empowers patient autonomy and shared decision-making during infant feeding discussions. |
| [*Infant feeding for people living with HIV in high resource settings: a multi-disciplinary approach with best practices to maximize risk reduction*](https://www.zotero.org/google-docs/?O1V7HP) | Powell  U.S.  2023 | To develop a structured shared decision-making process and protocol for successful implementation of breastfeeding for PLWH. | N/A | Narrative review/expert opinion | Framework for implementation of updated 2023 DHHS guidelines, including initiating conversations around infant feeding early, supporting the patient’s decision, and involving a multidisciplinary healthcare team to cover all aspects of care. |
| [*In support of breast-/chestfeeding by people with HIV in high-income settings.*](https://www.zotero.org/google-docs/?O1V7HP) | Powell  U.S.  2024 | To present the reasons for fully embracing  breastfeeding as a viable, safe infant feeding option for HIV-exposed infants in high-income settings, while acknowledging unanswered questions and the need to continually craft more nuanced clinical guidance. | N/A | Expert Opinion | Black, non-Hispanic women have the lowest rates of breastfeeding out of any racial group and are also disproportionately impacted by HIV. Systemic racism has led to many socio-cultural barriers to breastfeeding such as the belief that they prefer to formula feed, inflexible, low-wage jobs, short maternity leaves, financial pressure, lack of cultural normalization, and lack of support. |
| *Pregnancy in Women Living with HIV: Experience of IRCCS San Gerardo dei Tintori and a narrative review* | Ranzani  Italy  2025 | To describe the evolution of care for pregnant women  living with HIV and the current issues regarding ARV therapy, delivery, prophylaxis and  breastfeeding | N/A | Narrative review | Italian guidelines currently recommend against breastfeeding for women living with HIV. The authors highlight the need to measure maternal viral load more frequently among breastfeeding women living with HIV. |
| *Practice Variability in Uptake and Implementation of New U.S. DHHS Guidelines for Feeding of HIV-Exposed Infants* | Rozen Eisenberg  U.S.  2025 | To ascertain up-  take, practices, and perceptions surrounding new breastfeeding  guidelines. | Pediatric Infectious Disease Society members  N=83 clinicians at 69 centers | Cross-sectional | The authors found variable uptake of updated DHHS guidelines among pediatric infectious disease physicians. This variability may be explained by ethical considerations, ambiguity of guidance, and insufficient consensus data supporting breastfeeding. |
| *Breastfeeding with HIV in a high-income setting: equipoise and beyond - time to question the zero-risk policy* | Rudin  Switzerland  2025 | To summarize the literature regarding the risks of breastfeeding with HIV in high-income settings | N/A | Review | The authors discuss the concept of clinical equipoise, as well as the uniqueness of HIV in that unlike other diseases, any residual risk of HIV transmission has been deemed unacceptable by practitioners and governing bodies. This has led to hesitation to change guidelines even when they would eliminate other potential risks. |
| *HIV and Pregnancy: Navigating Complex Decision-making and Preventing Perinatal Transmission* | Short  U.S.  2025 | To examine the intersection of pregnancy and HIV, focusing on birthing person and fetal health outcomes, prevention of perinatal HIV transmission, and the latest advancements in treatment and care in the U.S. It highlights current guidelines, challenges in management, and future directions for improving  outcomes. | N/A | Review | There are no standardized recommendations regarding the frequency of viral load monitoring of the birthing parent or infant in the postpartum period. |
| *"My body my baby": a qualitative study examining drivers of infant-feeding choices among women living with HIV in Philadelphia, United States* | Zapata Vaca  U.S.  2025 | To examine personal and social determinants of feeding choice among PLHIV in the Philadelphia area just prior to the change in national guidelines in 2023 | Multiparous PLWH in Philadelphia, ages of 18–50 years old  N=28 | Qualitative | The authors found that participants were largely not given a choice during infant feeding decision-making. The most important factors for infant feeding decision-making was participants’ own knowledge regarding breastfeeding safety for their infant, and education from their providers. Community level factors such as influence from family and partner were less likely to influence decision-making. |
